# Case Report: Sudden death caused by methylene chloride poisoning

**DOI:** 10.3389/fphar.2024.1471744

**Published:** 2024-11-26

**Authors:** Ruikai Shang, Qiaoxin Tian, Yuru Liu, Hongyu Liu, Xiangxing Zhang, Mengdi Shi, Xiangdong Jian, Qilu Li

**Affiliations:** ^1^ Department of Poisoning and Occupational Diseases, Emergency Medicine, Qilu Hospital of Shandong University, Cheeloo College of Medicine, Shandong University, Jinan, Shandong, China; ^2^ Department of Occupational and Environmental Health, School of Public Health, Cheeloo College of Medicine, Shandong University, Jinan, Shandong, China; ^3^ School of Nursing and Rehabilitation, Cheeloo College of Medicine, Shandong University, Jinan, Shandong, China; ^4^ Department of Pharmacy, the Hospital of Shandong University, Cheeloo College of Medicine, Shandong University, Jinan, Shandong, China

**Keywords:** dichloromethane, acute poisoning, events, fractured ribs, cardiopulmonary resuscitation, cure

## Abstract

Dichloromethane is widely used as an organic solvent in aerospace, electronics, and medicine. Cases of poisoning caused by this substance are rare. Recently, we successfully treated a patient with an acute dichloromethane poisoning. During the production process, owing to pipeline leakage and lack of personal protection, the patient was poisoned by dichloromethane inhalation, fell from a height, and experienced a sudden cardiac arrest. After successful cardiopulmonary resuscitation, the patient was transferred to a local hospital for diagnosis and treatment and then to our hospital. After two visits to our hospital for systematic diagnosis and active treatment with fluid infusion, anti-infection therapy, glucocorticoids, ventilator-assisted respiration, chest strap fixation, and nutritional support, the patient achieved clinical recovery.

## 1 Introduction

Dichloromethane is a colorless, transparent, and volatile liquid, which is nonflammable and has a strong ether-like odor (Zheng et al., 2023). It is slightly soluble in water but completely soluble in ethanol and ether. Dichloromethane can be decomposed into hydrochloric acid, carbon dioxide, carbon monoxide, and phosgene when exposed to heat and humidity. It is widely used as an organic solvent in aerospace, electronics, pharmaceuticals, and medicine. Although dichloromethane has low toxicity, it has an anesthetic effect that can damage the central nervous and respiratory systems; in severe cases, it can cause cerebral edema and pulmonary edema.

We recently admitted a patient with acute dichloromethane poisoning, who fell from a height after inhaling dichloromethane and experienced a sudden cardiac arrest. After successful cardiopulmonary resuscitation, the patient was first sent to a local hospital for emergency treatment and then transferred to our department. After 35 days of hospitalization, the patient’s condition improved and he was discharged (Table 1). Two months later, the patient complained of chest pain and chest computed tomography (CT) revealed a high-density shadow on the lung. After rehospitalization and 9 days of treatment, the patient was discharged. Finally, at discharge and during the follow-up, he showed signs of full recovery.

**TABLE 1:** Biochemical blood test results of the patient.

Biochemical blood indicators	Normal values	Day 1	Day 3	Day 7	Day 14	Day 21
WBC (×10^9^/L)	3.50–9.50	16.23	10.59	11.37	11.86	7.40
NEU (%)	40.0–75.0	89.00	87.50	80.10	74.00	67.40
RBC (×10^12^/L)	4.30–5.80	4.30	4.04	4.08	3.04	3.61
HGB (g/L)	110.0–140.0	135	124	127	95	112
PLT (×10^9^/L)	125–350	121	149	263	440	299
ALB (g/L)	35–50	41.0	37.3	33.5	25.3	35.2
ALT (U/L)	9–50	48	22	206	49	39
AST (U/L)	15–40	60	17	84	23	19
CK (IU/L)	30–135	355	346	52	77	25
CK-MB (ng/mL)	0.3–4.0	7.10	2.30	0.70	3.50	1.3
BUN (mmol/L)	2.5–6.1	4.50	6.10	11.50	8.20	5.99
Cr (µmol/L)	46–106	70	62	58	49	57
LDH (IU/L)	120–246	436	415	339	282	226

ALB, albumin; ALT, alanine transaminase; AST, aspartate aminotransferase; BUN, blood urea nitrogen; CK, creatine kinase; CK-MB, creatine kinase isoenzyme; Cr, creatinine; LDH, lactate dehydrogenase; HGB, hemoglobin; NEU, neutrophils; PLT, platelets; RBC, red blood cells; WBC, white blood cells.

## 2 Case description

**FIGURE 1:**
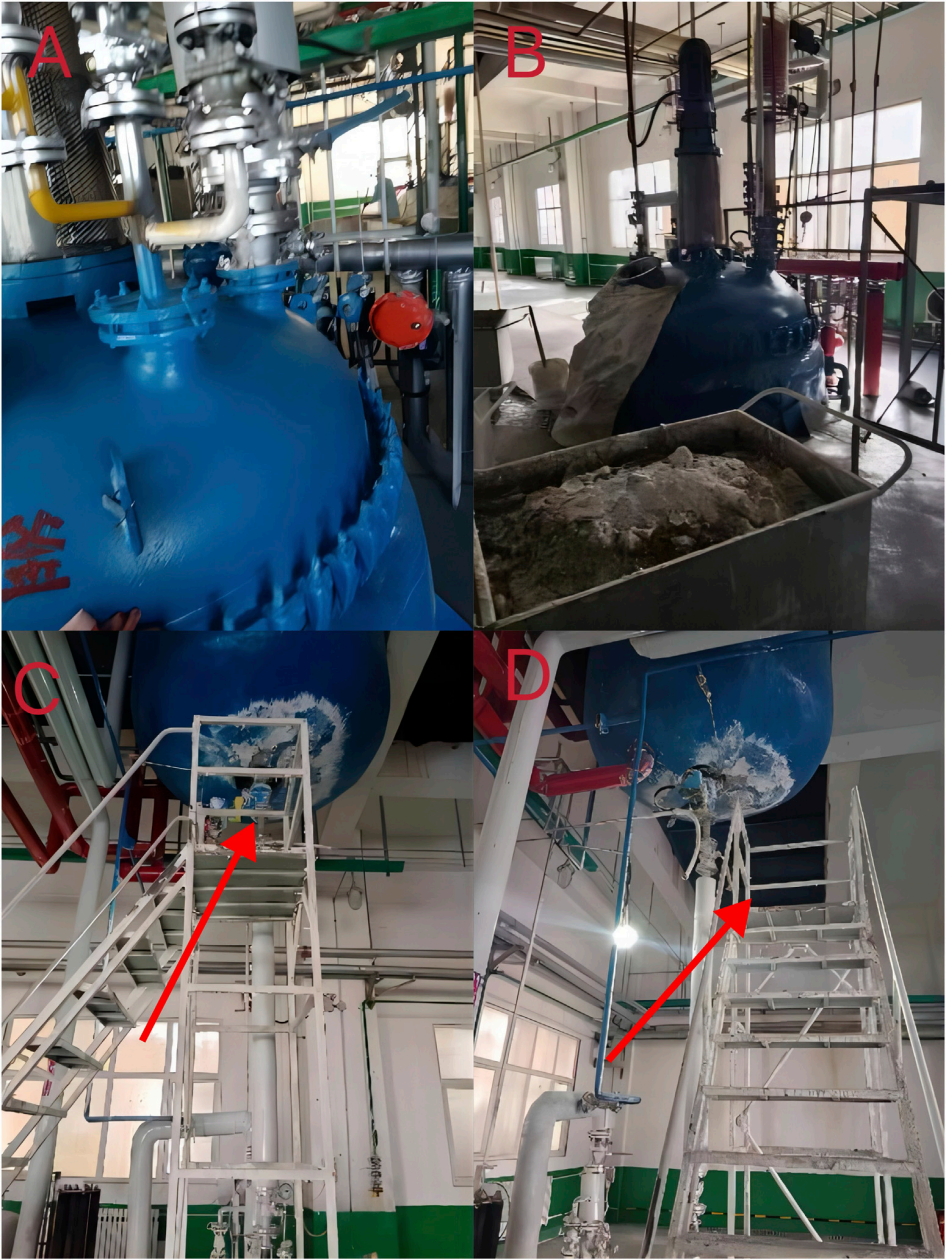
The factory working environment. **(A, B)** The upper end of the reactor; **(C, D)** The lower end of the reactor.

The patient was a 49-year-old man who was accidently exposed to dichloromethane on 16 October 2023, at a synthesis workshop at a pharmaceutical factory that used dichloromethane for a step in avastatin production. Figure 1 illustrates the upper (Figures 1A, B) and lower ends of the reaction kettle (Figures 1C, D). During the accidental leakage of dichloromethane on 16 October 2023, because of a miscommunication between operators and owing to the lack of a gas mask, the patient fainted and fell from a height after inhaling dichloromethane. Upon arrival, the emergency personnel found that the patient had a heartbeat but was in respiratory arrest. Arterial blood gas analysis showed that blood oxygen saturation was 71%, pH 7.2, partial pressure of oxygen 58 mmHg, partial pressure of carbon dioxide 45 mmHg, bicarbonate ion 20.1 mmol/L, lactic acid 6.2 mmol/L, and carboxyhemoglobin 6.4%.

**FIGURE 2:**
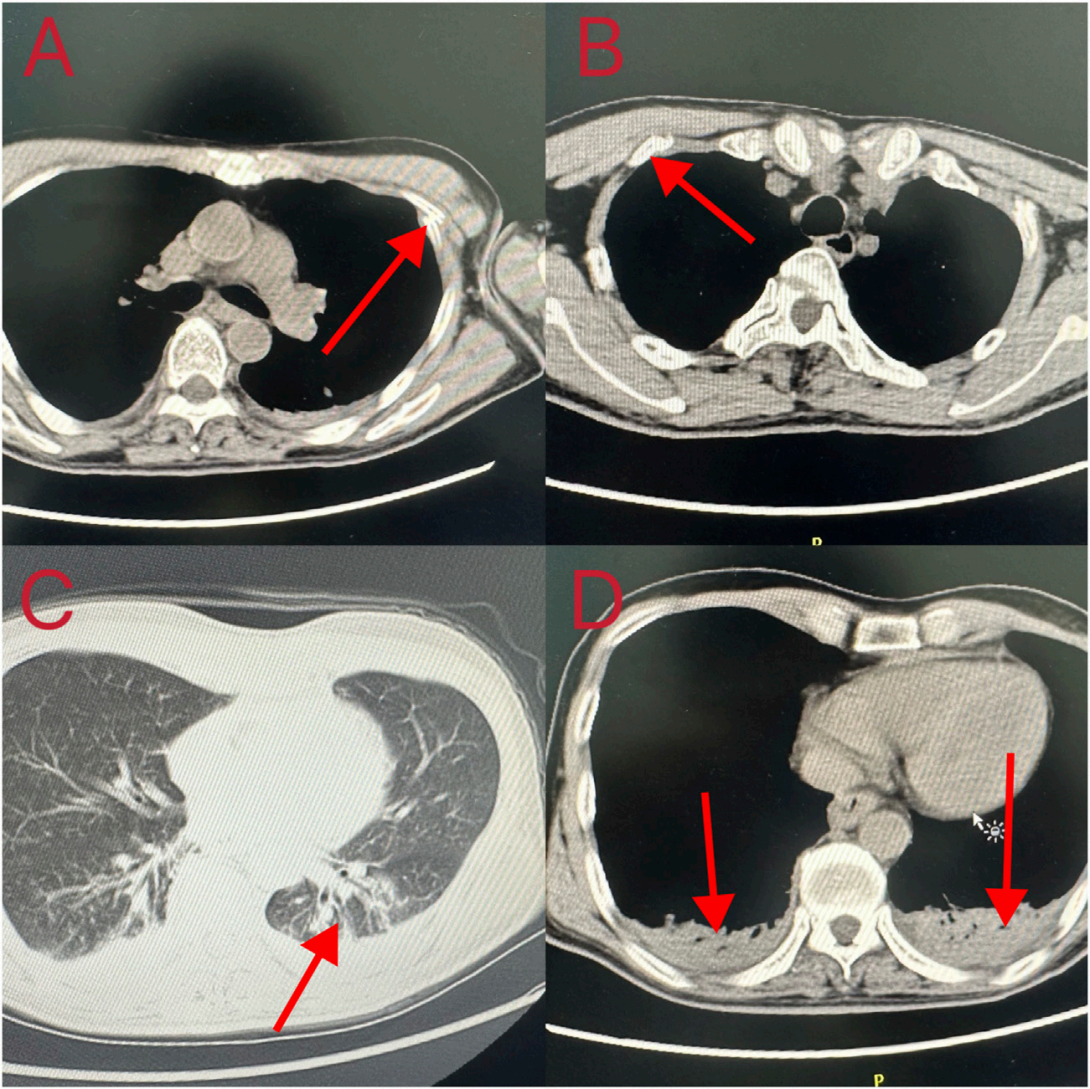
Computed tomography changes in the patient’s lungs. **(A, B)** Fractured ribs; **(C, D)** Double pneumonia and bilateral pleural effusion.

**FIGURE 3:**
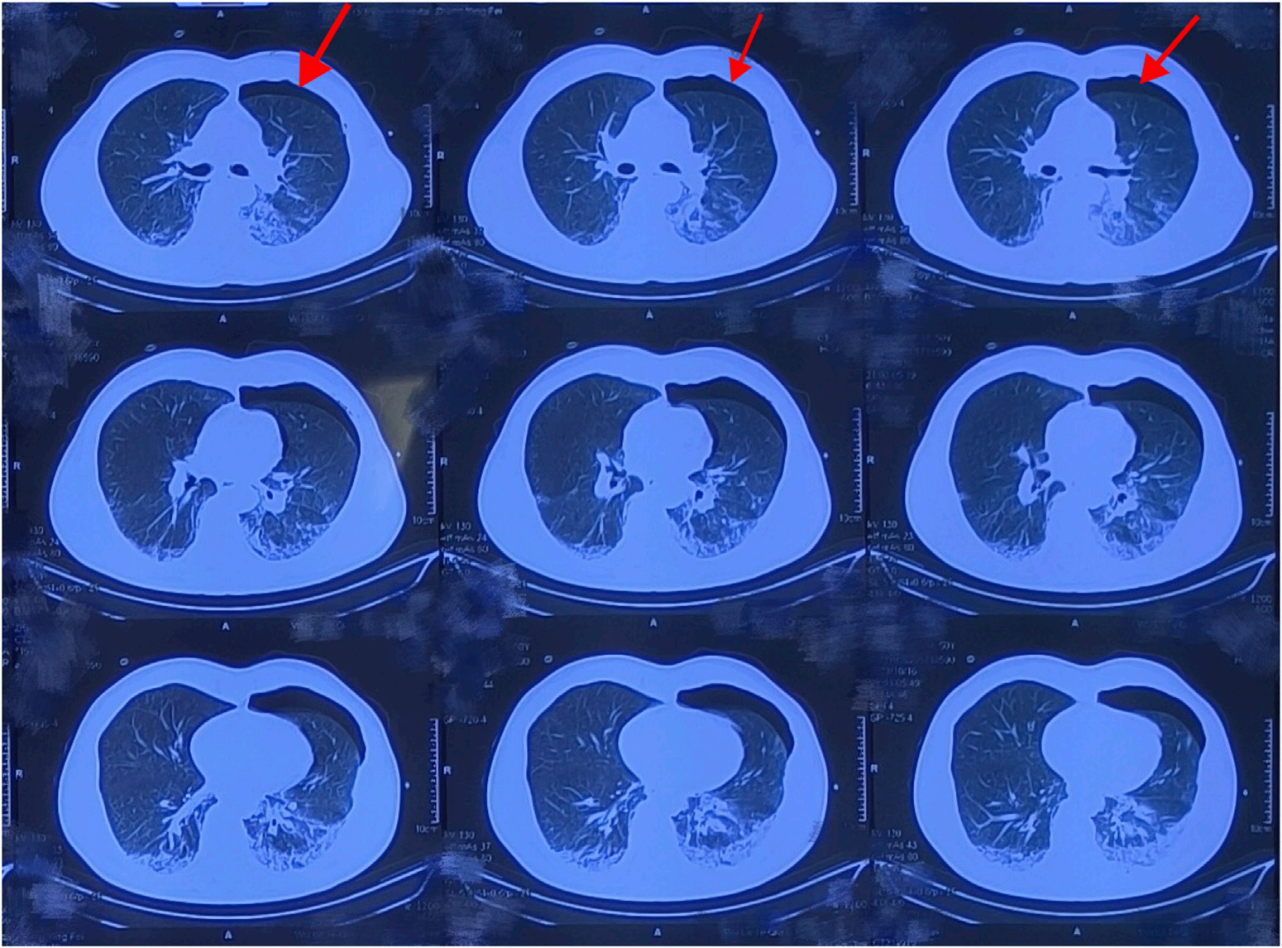
Computed tomography changes in the patient’s lungs. Pneumothorax.

**FIGURE 4:**
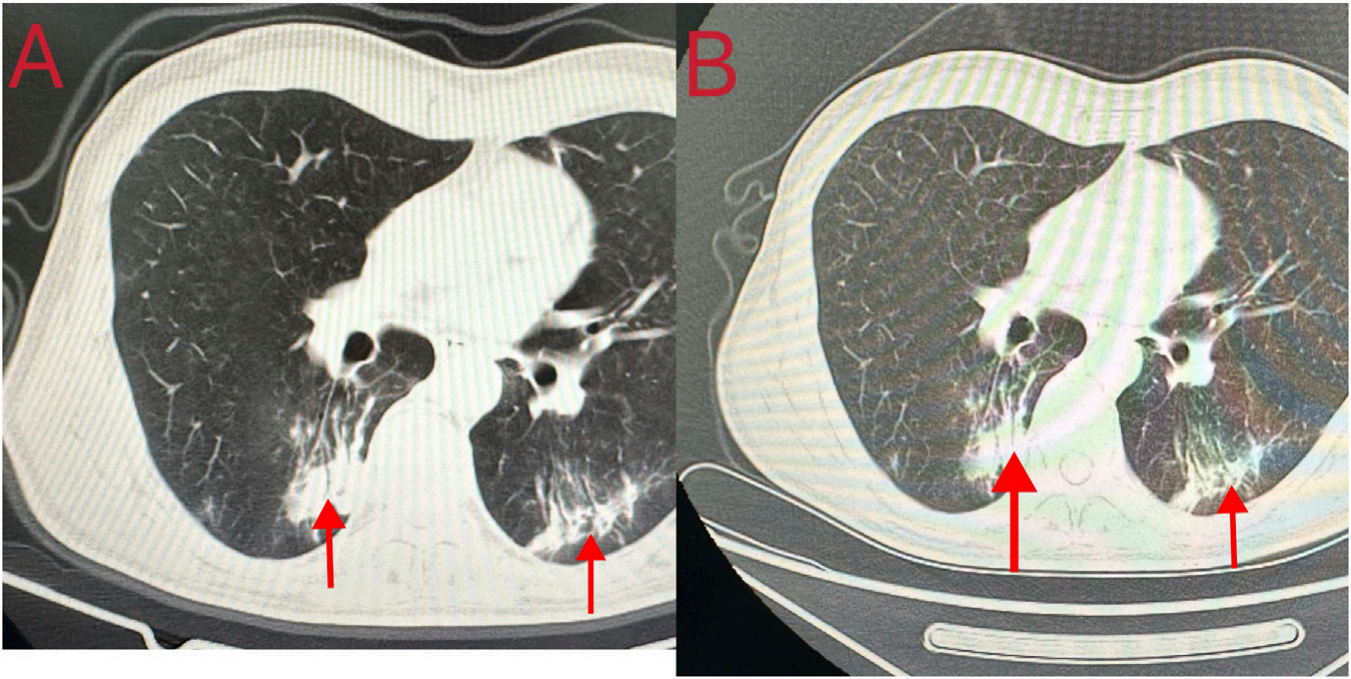
Chest computed tomography findings of the patient during the second adimission. **(A, B)** A high-density shadow in the lungs.

Cardiopulmonary resuscitation, oxygen inhalation, infusion, and other related treatments were urgently performed, and the patient was first sent to a local hospital for treatment. After admission, symptomatic treatments such as anti-infection, free radical scavenging, asthma relief, myocardial protection, anti-epilepsy, stomach protection, and fluid infusion were administered. After 2 days of hospitalization, he was transferred to our hospital for further diagnosis and treatment on the early morning of 18 October 2023. Physical examination on admission revealed a body temperature of 37.1°C, pulse rate of 92 beats/min, respiratory rate of 20 breaths/min, and blood pressure of 100/86 mmHg. He was intubated and placed on ventilator-assisted ventilation and was in a coma. The pupils were 4 mm in diameter and unresponsive to light. Bilateral breath sounds were coarse and no dry or wet rales were heard. The rhythm was normal and no pathological murmur was heard in any valve area. The abdomen was flat and soft, and the liver and spleen under the ribs were not palpable. Physiological reflex was absent and a pathological reflex was not elicited. Electrocardiogram examination at admission revealed the following: sinus rhythm, abnormal lower wall ST-T, possibly due to myocardial ischemia. Auxiliary test results were as follows: blood gas analysis: arterial partial pressure of oxygen 201.5 mmHg; arterial partial pressure of carbon dioxide 49.3 mmHg; white blood cell count 16.23 × 10^9^/L (3.5–9.5 × 10^9^/L), neutrophil ratio 89% (40%–75%); and platelet count 121 × 10^9^/L (125–350 × 10^9^/L). Laboratory blood test results were as follows: alanine aminotransferase 48 U/L (9–50 U/L), aspartate aminotransferase 60 U/L (15–40 U/L), serum myoglobin 114 ng/mL (0–70 ng/mL), creatine kinase isoenzyme 7.10 ng/mL (0.3–4 ng/mL), serum high-sensitivity troponin I 170.85 (<17.5 ng/L), N-terminal pro-brain natriuretic peptide 1,387 pg/mL (emergency ≤300 pg/mL), interleukin-6 63.50 pg/mL (0–7 pg/mL), and interleukin-1b 6.15 pg/mL (0–5 pg/mL). Brain CT and magnetic resonance imaging showed no apparent abnormalities. Chest CT revealed double pneumonia, bilateral pleural effusion, multiple fractures of the left 1st to 6th ribs, fractures of the right 2nd and 3rd ribs, severe respiratory artifacts (Figure 2), and left pneumothorax (Figure 3). The admission diagnoses were acute dichloromethane poisoning, hemopneumothorax, and rib fracture. After admission, the patient was actively treated with anti-infection therapy, reduce organ edema, glucocorticoids, ventilator-assisted respiration, chest strap fixation, and nutritional support. On the 7th day after admission, the patient’s blood test results revealed a decrease in white blood cell count 11.37 × 10^9^/L (3.5–9.5 × 10^9^/L) and neutrophil ratio 80.1% (40%–75%), and increased levels of alanine aminotransferase 206 U/L (9–50 U/L) and aspartate aminotransferase 84 U/L (15–40 U/L). On the 14th day of admission, routine blood, liver, and kidney function tests showed no obvious abnormalities, and the tracheal tube was removed. On the 29th day of admission, electromyography showed lesions of the right median nerve in the wrist and lesions of the right superficial peroneal nerve. The electroencephalogram showed no abnormalities. After 35 days of treatment, the patient’s condition improved and he was discharged. On 26 December 2023, the patient complained of chest pain and underwent a reexamination. Chest CT showed a high-density shadow in the lungs (Figure 4). After 9 days of comprehensive anti-infection treatment, the patient’s condition improved, and he was discharged. Follow-up after discharge indicated the absence of sequelae. The patient was transferred from his original job and engaged in other work.

This study was approved by the Ethics Committee of Qilu Hospital of Shandong University.

## 3 Discussion

Dichloromethane, when inhaled, can cause serious pulmonary and brain edema (Snyder et al., 1992). Dichloromethane poisoning occurs via inhalation, oral administration, or skin contact, and exposure to dichloromethane is mostly occupational (Hoang et al., 2021). There are two metabolic pathways in the body: the main pathway is the cytochrome P450 enzyme pathway that produces carbon monoxide and carbon dioxide; the second is the glutathione transferase pathway that produces carbon dioxide (Manno et al., 1992). Dichloromethane is continuously metabolized to produce carbon monoxide, the concentration of which may increase to toxic levels in the body. The toxicological mechanism of alkyl halides such as dichloromethane on the body is that they rapidly penetrate the cell membrane and lead to intracellular poisoning (Mahmud and Kales, 1999). One study has shown that dichloromethane can damage liver structure and function in mice via the generation of free radicals catalyzed by endoplasmic reticulum hydroxylases in hepatocytes (Mizutani et al., 1988). Free radicals can covalently bind to macromolecules in cells and cell membranes, inactivate enzymes, cause lipid peroxidation of cellular components, alter cell membrane integrity, rupture lysosomes, and damage the mitochondria. The mechanism of its toxic effect on the heart may be related to the production of carbon monoxide in the body, which reduces the ability of blood to supply oxygen to the myocardium, or may be related to its direct toxicity to the heart. When dichloromethane is inhaled, it is exposed to heat and moisture in the respiratory tract and can be broken down into hydrochloric acid and phosgene, which have a stimulating effect on the respiratory tract and lungs and can lead to pulmonary edema in severe cases (Snyder et al., 1992).

Acute poisoning mainly leads to inhibition of the central nervous system. The main symptoms include dizziness, headache, nausea, vomiting, cough, and fatigue. In severe cases, dyspnea, disturbance of consciousness, and convulsions may occur, which generally appear several hours after poisoning, gradually aggravate in 1–2 days, peak in 3–5 days, gradually reduce after 1 week, and recover after 2–4 weeks (Rioux and Myers, 1988). Because the concentration of carboxyhemoglobin will increase in moderate to severe poisoning (Chang et al., 1999), blood carboxyhemoglobin level can be used as a diagnostic reference when it is increased (Raphael et al., 2002). In this report, the patient presented with metabolic acidosis and hyperlactatemia; and elevated lactate levels are often related to tissue cell hypoxia and hypoperfusion. The manifestations of central nervous system damage may be related to the paralytic effects of toxic dichloromethane substances on the central nervous system. Simultaneously, carbon monoxide produced by dichloromethane metabolism also has certain deleterious effects on the nervous system. The patient fell from a height and had multiple rib and sternum fractures; he also showed abnormal electrocardiographic findings and levels of serum troponin, myocardial enzymes, B-type natriuretic peptide and other biological indicators. Given the patient’s condition and situation, the patient was believed to have had a fall-related cardiac contusion. Chest trauma is an important risk factor for cardiac contusion. Cardiac contusion is a difficult clinical condition to diagnose, and there is neither a diagnostic test with high specificity and sensitivity nor a consensus on this issue. We recommend screening patients with cardiac contusion using continuous electrocardiographic monitoring and troponin levels (Sağlam Gürmen and Tulay, 2022).

There is no specific antidote for dichloromethane poisoning, and clinical treatment is mainly comprehensive. The first step is to remove the patient from the scene as quickly as possible. Therefore, the breathing conditions of patients with inhalation poisoning should be closely monitored. If coughing or dyspnea occurs, oxygen should be administered immediately, and the carboxyhemoglobin concentration should be measured. Oxygen can be administered using a nasal catheter or mask, and if necessary, through tracheal intubation or tracheotomy to rapidly increase the oxygen partial pressure in the body; dynamic blood gas analysis can be performed over time (Leikin et al., 1990).

This was an interesting and rare toxicological poisoning event. The patient had the following characteristics: inhalation of a large dose of dichloromethane, fall from a height, on-site cardiopulmonary resuscitation, multiple bilateral rib fractures, pneumonia; respiratory failure, tracheal intubation, mechanical ventilation, and disorders of consciousness. The key factors contributing to the successful rescue of the patient were the quick extraction of the patient from the toxic environment, on-site cardiopulmonary resuscitation, establishment of a ventilation airway and administration of mechanical ventilation, effective infection control, rational use of glucocorticoids, correct treatment of multiple rib fractures, and standardized nutritional support. This poisoning incident serves as a reminder to strengthen the emergency training on poisoning emergencies for toxic and harmful operations, strictly follow the operating rules, and enforce personal protection and workers’ awareness of personal protection. The limitation of this study is that we were unable to obtain blood and urine samples and on-site occupational hygiene investigation data at the time of the patient’s poisoning. The patient was transferred to our hospital after receiving treatment in a local hospital for 2 days. Toxicant analysis of the patient indicated the absence of methylene chloride in the blood and urine.

In conclusion, as there is currently no specific antidote for dichloromethane poisoning, the key lies in its prevention. The concentration of dichloromethane in the working environment can be monitored in real-time. In the event of poisoning in China, it should be treated quickly and the emergency number 120 should be called for a quick rescue to avoid delays in treatment.

## Data Availability

The original contributions presented in the study are included in the article/supplementary material, further inquiries can be directed to the corresponding authors.

## References

[B1] Chang Y. L. Yang C. C. Deng J. F. Ger J. Tsai W. J. Wu M. L. (1999). Diverse manifestations of oral methylene chloride poisoning: report of 6 cases. J. Toxicol. Clin. Toxicol. 37, 497–504. 10.1081/clt-100102442 10465248

[B2] Hoang A. Fagan K. Cannon D. L. Rayasam S. D. G. Harrison R. Shustermanet D. (2021). Assessment of methylene chloride-related fatalities in the United States, 1980-2018. JAMA Intern. Med. 181, 797–805. 10.1001/jamainternmed.2021.1063 33871539 PMC8056315

[B3] Leikin J. B. Kaufman D. Lipscomb J. W. Burda A. M. Hryhorczuk D. O. (1990). Methylene chloride: report of five exposures and two deaths. Am. J. Emerg. Med. 8, 534–537. 10.1016/0735-6757(90)90158-v 2222600

[B4] Mahmud M. Kales S. N. (1999). Methylene chloride poisoning in a cabinet worker. Environ. Health Perspect. 107, 769–772. 10.1289/ehp.99107769 10464079 PMC1566447

[B5] Manno M. Rugge M. Cocheo V. (1992). Double fatal inhalation of dichloromethane. Hum. Exp. Toxicol. 11, 540–545. 10.1177/096032719201100617 1361146

[B6] Mizutani K. Shinomiya K. Shinomiya T. (1988). Hepatotoxicity of dichloromethane. Forensic Sci. Int. 38, 113–128. 10.1016/0379-0738(88)90015-1 3192131

[B7] Raphael M. Nadiras P. Flacke-Vordos N. (2002). Acute methylene chloride intoxication—a case report on domestic poisoning. Eur. J. Emerg. Med. 9, 57–59. 10.1097/00063110-200203000-00013 11989499

[B8] Rioux J. P. Myers R. A. (1988). Methylene chloride poisoning: a paradigmatic review. J. Emerg. Med. 6, 227–238. 10.1016/0736-4679(88)90330-7 3049777

[B9] Sağlam Gürmen E. Tulay C. M. (2022). Attention: cardiac contusion. Ulus. Travma Acil Cerrahi Derg. 28, 634–640. 10.14744/tjtes.2021.11290 35485460 PMC10442995

[B10] Snyder R. W. Mishel H. S. Christensen G. C. (1992). Pulmonary toxicity following exposure to methylene chloride and its combustion product, phosgene. Chest 101, 860–861. 10.1378/chest.101.3.860 1541163

[B11] Zheng X. Zhuang Y. Liang D. Wu N. Wei T. Kang A. (2023). Dichloromethane-induced poisoning from acrylic paint cleaner – shenzhen city, guangdong province, China, 2023. China CDC Wkly. 5, 966–969. 10.46234/ccdcw2023.182 38025514 PMC10652081

